# ICA model order selection of task co-activation networks

**DOI:** 10.3389/fnins.2013.00237

**Published:** 2013-12-10

**Authors:** Kimberly L. Ray, D. Reese McKay, Peter M. Fox, Michael C. Riedel, Angela M. Uecker, Christian F. Beckmann, Stephen M. Smith, Peter T. Fox, Angela R. Laird

**Affiliations:** ^1^Research Imaging Institute, University of Texas Health Science Center, San AntonioTX, USA; ^2^Olin Neuropsychiatry Research Center, Institute of Living, Hartford Hospital, HartfordCT, USA; ^3^Department of Psychiatry, Yale University School of Medicine, New HavenCT, USA; ^4^MIRA Institute for Biomedical Technology and Technical Medicine, University of TwenteEnschede, Netherlands; ^5^Department of Clinical Neurology, Oxford Centre for Functional MRI of the Brain (FMRIB Centre), University of OxfordOxford, UK; ^6^Research Service, South Texas Veterans Administration Medical Center, San AntonioTX, USA; ^7^Department of Physics, Florida International University, MiamiFL, USA

**Keywords:** meta-analysis, co-activations, BrainMap, intrinsic connectivity networks, functional brain networks, functional connectivity, resting state networks, independent component analysis

## Abstract

Independent component analysis (ICA) has become a widely used method for extracting functional networks in the brain during rest and task. Historically, preferred ICA dimensionality has widely varied within the neuroimaging community, but typically varies between 20 and 100 components. This can be problematic when comparing results across multiple studies because of the impact ICA dimensionality has on the topology of its resultant components. Recent studies have demonstrated that ICA can be applied to peak activation coordinates archived in a large neuroimaging database (i.e., BrainMap Database) to yield whole-brain task-based co-activation networks. A strength of applying ICA to BrainMap data is that the vast amount of metadata in BrainMap can be used to quantitatively assess tasks and cognitive processes contributing to each component. In this study, we investigated the effect of model order on the distribution of functional properties across networks as a method for identifying the most informative decompositions of BrainMap-based ICA components. Our findings suggest dimensionality of 20 for low model order ICA to examine large-scale brain networks, and dimensionality of 70 to provide insight into how large-scale networks fractionate into sub-networks. We also provide a functional and organizational assessment of visual, motor, emotion, and interoceptive task co-activation networks as they fractionate from low to high model-orders.

## Introduction

Independent component analysis (ICA) offers a methodology for investigating functional brain connectivity of intrinsic neural networks in human neuroimaging data (Beckmann, 2012; Calhoun and Adali, 2012). This exploratory approach provides an alternative to hypothesis-driven connectivity techniques (e.g., seed-based correlation analyses; Biswal et al., 1995) by identifying independently distributed spatial patterns depicting source processes in multivariate data (McKeown and Sejnowski, 1998; Biswal and Ulmer, 1999; Hyvärinen and Oja, 2000; Beckmann et al., 2005; Calhoun et al., 2008). Over the last few years, improvements in ICA techniques (Long et al., 2007; Sohn et al., 2012), as well as their widespread application to functional magnetic resonance imaging (FMRI) data in healthy (Damoiseaux et al., 2006; Kiviniemi et al., 2009; Biswal et al., 2010; Allen et al., 2011; Mowinckel et al., 2012) and clinical populations (Greicius et al., 2004, 2007; Seeley et al., 2007; Sorg et al., 2007; Jafri et al., 2008; Wolf et al., 2008; Mohammadi et al., 2009; Zhang et al., 2009; Qi et al., 2010; Li et al., 2012; Zhou et al., 2012), have substantially enhanced our knowledge of resting state functional connectivity (Ding et al., 2011; Allen et al., 2012; Calhoun et al., 2012; Jones et al., 2012; Bridwell et al., 2013).

Recent evidence has shown that these intrinsic connectivity networks (ICNs) can also be extracted from a large neuroimaging database of task-based co-activation patterns (Fox and Lancaster, 2002) both by region-seeding (Toro et al., 2008) and by ICA (Smith et al., 2009; Laird et al., 2011a). An advantage of studying task co-occurrence networks derived from the BrainMap database is that the BrainMap behavioral taxonomy (Fox et al., 2005; Laird et al., 2005) for describing functional neuroimaging tasks allows quantitative classification and relatively automated interpretation of the functional significance of these networks (Laird et al., 2011b). This was demonstrated by Smith et al. (2009) when the behavioral metadata of BrainMap-based ICNs was used to describe the functional nature of corresponding resting-state networks, and again in Laird et al. (2011a) when providing a full functional explication of BrainMap ICA networks at a standard low model order (*d* = 20). This use of BrainMap metadata allows for a substantially more rich characterization of ICNs than previously possible. The BrainMap approach differs from analysis of data acquired during the task-free resting state, where network functional characterization relies heavily on subjective visual inspection to draw upon similarity to published task-based results.

In both resting state FMRI and task-based meta-analytic data, ICA is carried out at a given model order to identify a set of *d* (*d* = dimensionality) spatial components and their associated time courses or database weighting vectors. Although automated methods for model order estimation in resting state FMRI data have been developed (Beckmann and Smith, 2004; Himberg et al., 2004; Li et al., 2007), these methods can be somewhat arbitrary and usually depend upon a number of factors (e.g., field strength, number of time points, number of subjects, and data quality), in which their application may not be robust enough to rely on for all implementations of ICA. Model order selection has a significant impact on the spatial organization of resultant ICNs since networks at low model orders are fractionated into sub-networks as model order increases (Kiviniemi et al., 2009; Smith et al., 2009; Abou Elseoud et al., 2010). Across varying dimensionalities, ICA of resting state FMRI data has consistently revealed a hierarchical modularity to functional brain organization, in agreement with results from graph theoretic approaches (He et al., 2009; Meunier et al., 2009). Our focus in the present study is how ICA model order may be leveraged in analyses of task co-activation networks to more fully investigate this hierarchical modularity.

The functional assessment of network fractionation across task-based ICA components is also strongly influenced by model order selection, since this parameter affects spatial topography, which in turn impacts classification of the behavioral domains and paradigms associated with the networks at any given model order (Smith et al., 2009). Previous analysis of BrainMap co-activation networks was performed at model orders of 20 and 70 components, to simplify comparisons with prior analyses of resting state FMRI data at these dimensionalities (Smith et al., 2009; Laird et al., 2011a); however, differentiation of preferred model orders is needed before a more complete examination of network fractionation can be made. Here, we present a data-driven method for identifying BrainMap-based ICA decompositions that provide increased explication for functional decoding of task co-activation networks. In addition, we examine the functional organization as well as the topology of the resultant ICNs as they fractionate from low to high model orders.

## Materials and methods

### Generation of intrinsic connectivity network maps

**Figure 1:**
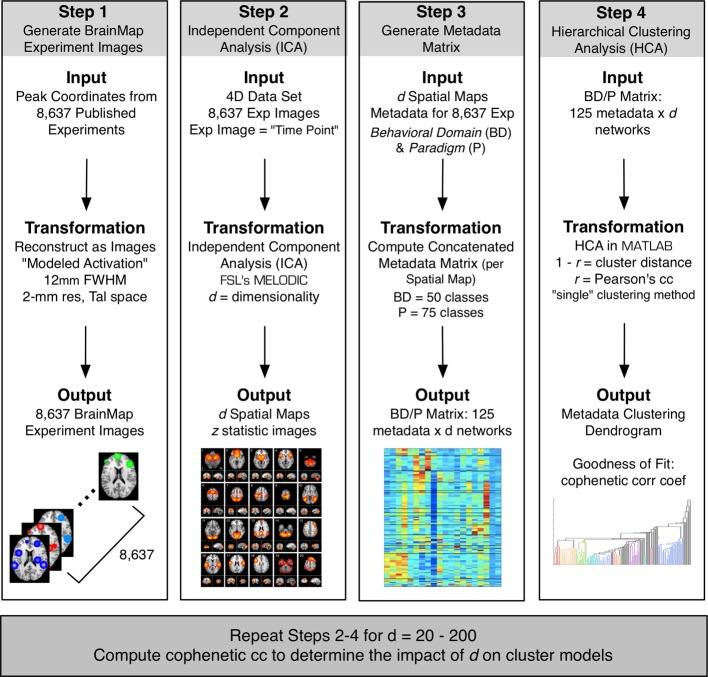
**The BrainMap ICA processing stream included four steps**. Step 1: Peak activation coordinates from 8,637 experiments in the BrainMap database were smoothed at a FWHM of 12 mm to create a 4D modeled activation map (space × experiment ID). Step 2: ICA was applied to the 4D data using FSL's MELODIC at a model order of *d* to create a set of *d* spatial components. Step 3: Metadata matrices were created at each *d* weighting how strongly each component related to the behavioral domain and paradigm metadata classes in BrainMap (125 metadata classes × *d* components). Step 4: HCA was performed on metadata matrices, and the CC*_c_* of the resultant dendrogram was computed to determine the fit of the clustering for that model order. These 4 steps were repeated for a range of *d*, from 20 to 200 in intervals of 10.

Peak activation coordinates were extracted from 8,637 functional neuroimaging experiments archived in the BrainMap database (Fox and Lancaster, 2002; Laird et al., 2005, 2009, 2011a; http://brainmap.org) and smoothed (*FWHM* = 12 mm) to create modeled activation images. Spatial ICA was applied to this 4D dataset (space × experiment ID) using MELODIC (multivariate exploratory linear optimized decomposition into independent components; Beckmann et al., 2005) provided by FSL (FMRIB Software Library; Smith et al., 2004; Woolrich et al., 2009; Jenkinson et al., 2012). ICA was performed at multiple dimensionalities, creating sets of 20–200 independent components, at intervals of 10 (Figure 1). At each model order, ICA maps were converted to *z* statistic images via a normalized mixture model fit, thresholded at *z* > 4, and viewed on a Talairach space template image (Kochunov et al., 2002).

### Metadata matrices

Each set of published coordinates of experimental task activation locations in the BrainMap database is associated with a highly organized taxonomy of metadata that describes information about the scanned subjects (e.g., behavioral conditions, experiment design, and imaging analysis parameters). Behavioral domain and paradigm class are two metadata fields that have been found to contain the most explanatory power of functional characterization per component (Laird et al., 2011a). There are currently 75 available paradigms classifying the experimental task employed during functional image acquisition (Turner and Laird, 2012), and 50 behavioral domains describing the categories of cognitive processes isolated by an experimental contrast (Fox et al., 2005; http://brainmap.org/scribe). To systematically identify the paradigms and behaviors associated with BrainMap ICA components at a given model order, a matrix (125 metadata classes × *d* components) was created for each ICA decomposition, which quantifies the relationship between a given component and the behavioral domains or paradigms (Smith et al., 2009; Laird et al., 2011a). In doing so, the matrix *M* was computed, which is an *e* × *d* matrix whose *e* rows (one for each experiment) and *d* columns (one for each ICA component) describe the weightings of each component for each of the original activation images,

(1) $$M=V_{d}M_{d}$$

where *V_d_* includes the *d* largest singular values of the “temporal” (experiment ID) modes and *M_d_* is the mixing matrix of size *d* × *d*. We then extracted the *n* (behavioral domain and paradigm metadata classes) × *e* (experiment ID) matrix *P* from the BrainMap archive to form the final matrix of metadata classes vs. ICA components,

(2) $$P_{d}=PM$$

The resultant metadata matrix measures how strongly each behavioral domain and paradigm relate to the individual components at a specified model order, which informs interpretation of the functional properties for the ICNs. Repeating these steps (Figure 1, Steps 2–4) across multiple ICA model orders from *d* = 20 to 200 yielded 19 ICA decompositions, and hence 19 metadata matrices, provided a quantitative representation of the dynamic functional nature of ICNs across a wide range of model orders.

### Hierarchical clustering analysis

In our previous study, we established that hierarchical clustering analysis (HCA) of the metadata matrix at *d* = 20 provided a data-driven method for establishing groupings of similar tasks and behaviors (Laird et al., 2011a). Here, we propose that HCA be used to introduce a metric for discriminating across ICA model order. As described above, a metadata matrix was created for a specified model order that assigned weights of the strength of each functional metadata class to each ICN. HCA was then performed on this metadata matrix, yielding a dendrogram that allowed visualization of groups of similar metadata classes for that model order, where each branch in the dendrogram represents a single paradigm or behavioral domain (Figure 1, Step 4). We then computed the cophenetic correlation coefficient (CC*_c_*) for the dendrogram:

(3) $$CC_{c}=\frac{\sum_{i<J}\left(Y_{ij}-y\right)(Z_{ij}-z)}{\sqrt{\sum_{i<J}{\left(Y_{ij}-y\right)}^{2}\sum_{i<J}{\left(Z_{ij}-z\right)}^{2}}}$$

where *Y_ij_* is the distance between objects *i* and *j* (where *i* and *j* are the indices of the values in the *d* × 125 metadata matrix) in *Y*, *Z_ij_* is the cophenetic distance between objects *i* and *j* in *Z* (the height of the node at which these two points are first joined together in the dendrogram), and *y* and *z* are the averages of *Y* and *Z,* respectively. By definition, the cophenetic correlation coefficient for a cluster tree is the linear correlation coefficient between the cophenetic distances obtained from the tree, and the original distances (or dissimilarities) used to construct the tree (Sokal, 1962), where values close to 1 indicate a high-quality solution. The CC*_c_* of metadata clustered dendrograms were recorded across model order, as opposed to network clustered dendrograms, to maintain a comparison of models with a consistent number of branches.

In this context, we employed the CC*_c_* to assess how well each set of *d* clustering results reflected its corresponding metadata matrix for a given model order. We hypothesized that computing the cophenetic correlation coefficient across a range of models orders would provide insight into how well the distributions of functional properties fit co-activation networks across different ICA decompositions. This method was based on the assumption that high CC*_c_* values results in more informative ICA decompositions of task-based intrinsic connectivity networks. HCA was performed in MATLAB (The Mathworks Inc., Natick, Massachusetts) on the 19 metadata matrices (one for each model order) using the *single* linkage algorithm with 1 −r as the distance between clusters, where *r* is the Pearson's correlation coefficient, and unthresholded cluster distances, similar to the procedure developed by Laird et al. (2011a).

## Results

### Significant mean volume and z-Score of ICNs

**Figure 2:**
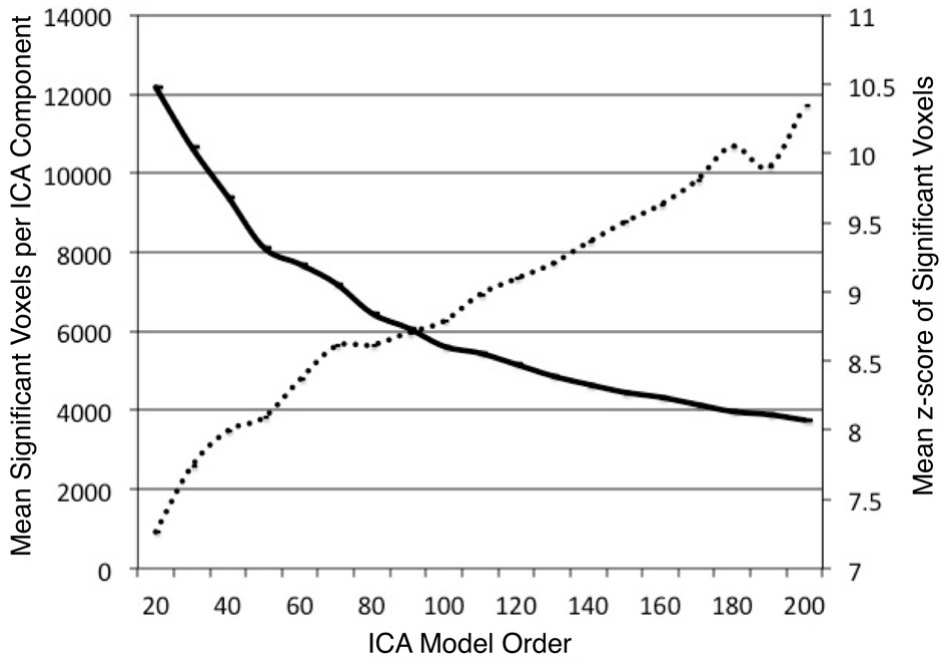
**The mean number of significant voxels (*z* > 4) of ICA components decreased as model order increased (solid, bold), while the mean *z*-score of significant voxels in ICA components increased almost linearly (*R*^2^ = 0.975) as model order increased (dashed line)**.

ICA was performed on the BrainMap database to identify task co-activation networks at model orders of *d* = 20–200, in intervals of 10. We investigated the impact of model order on the BrainMap-based ICA spatial component maps. Across each dimensionality, the average number of significant voxels and the average *z*-score of significant voxels were computed for each thresholded component (*z* > 4). As expected, these values showed substantial changes as a result of altering model order. The average number of significant voxels per component showed a logarithmic decrease (*R*^2^ = 0.996) as model order increased (Figure 2). This result was expected since increasing model order forces ICA to separate or fractionate large spatial components into smaller components. The average component z-score increased nearly linearly (*R*^2^ = 0.975) as model order increased. Our overall observation was that as the number of components increased, the average volume of individual components decreased, resulting in a higher concentration of significant activity spanning across fewer voxels. Similar results were observed by Abou Elseoud et al. (2010) when investigating ICA model order of intrinsic connectivity networks in resting state FMRI data.

### Hierarchical clustering of brainmap component metadata

**Figure 3:**
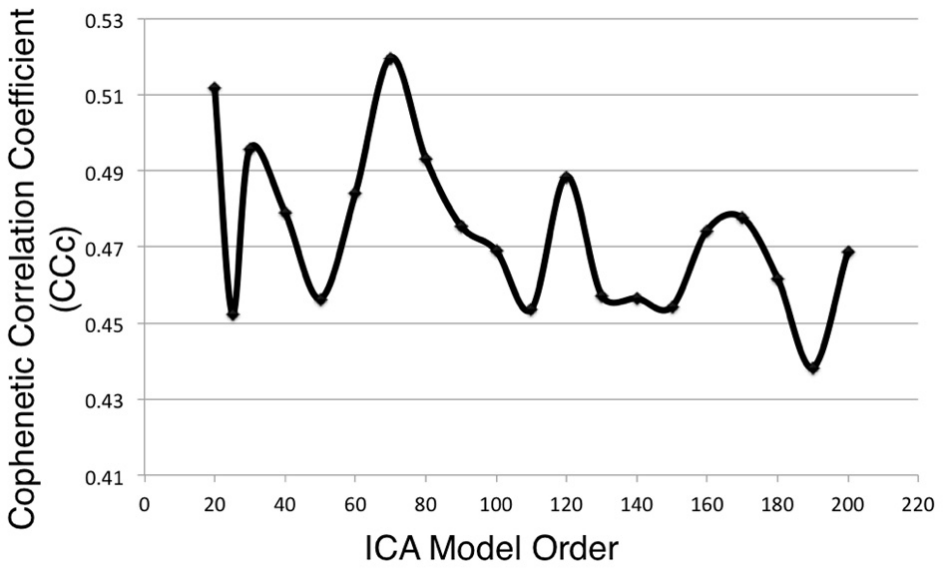
**Cophenetic correlation coefficients (CC*_c_*) were computed for the BrainMap metadata matrices across ICA model orders**. CC*_c_* values indicate how well the HCA results fit the corresponding BrainMap metadata. The ICA model orders yielding the two highest CC*_c_* values were generated with metadata from the *d* = 20 (CC*_c_* = 0.5119) and *d* = 70 (*CC_c_* = 0.5198) decompositions.

**Figure 4:**
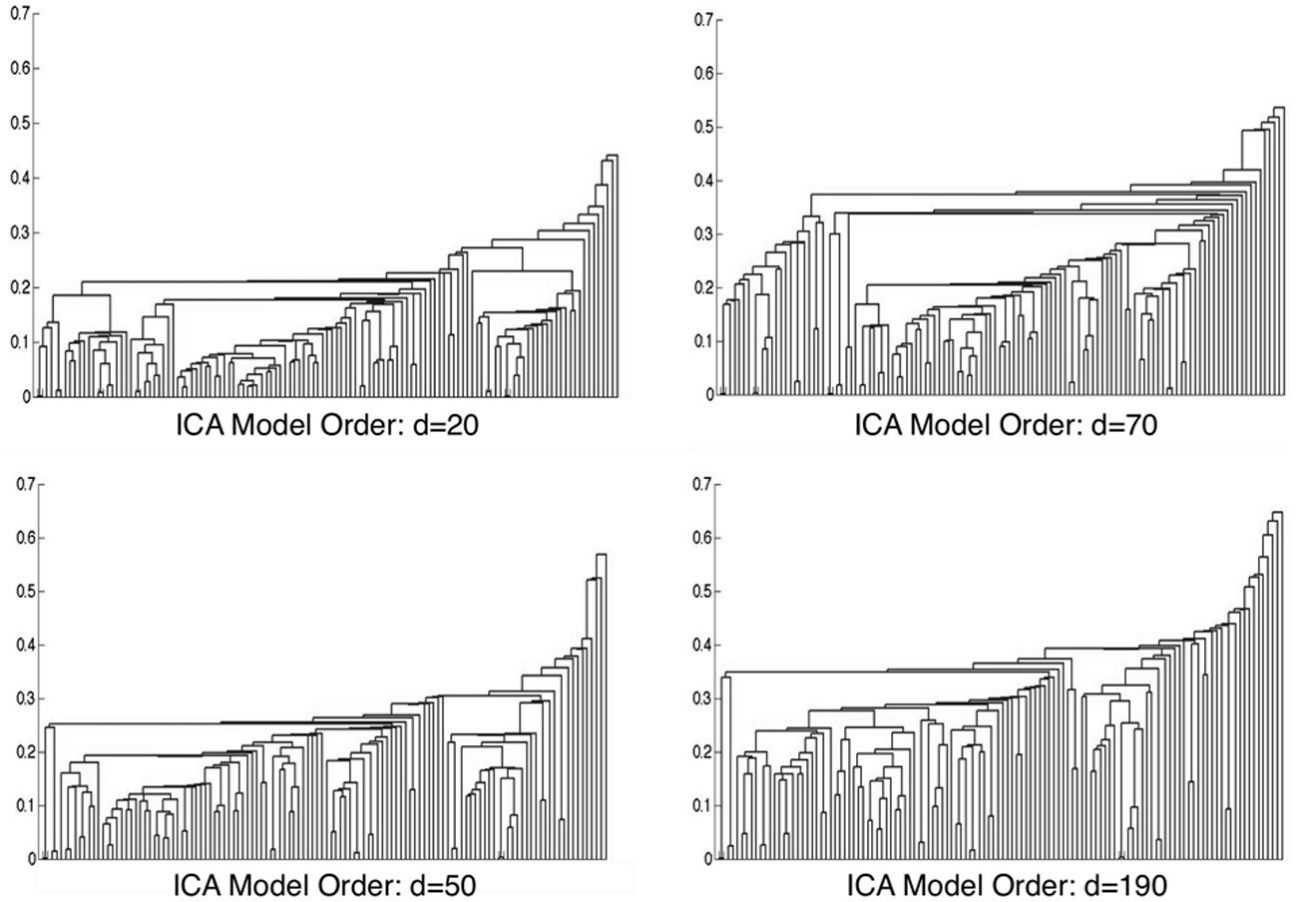
**Hierarchical clustering dendrograms are shown for ICA model orders of 20, 50, 70, and 190**. Model orders of 20 and 70 resulted in the two highest CC*_c_* values, while *d* = 50 and *d* = 190 resulted in the lowest CC*_c_* values. The dissimilarity scale (y-axis) of each dendrogram indicates how strongly the behaviors and paradigms were found to cluster together. High branching points along the dissimilarity axis in the *d* = 50 and *d* = 190 dendrograms indicate less agreement across variables, whereas lower branching points in the *d* = 20 and *d* = 70 networks indicate a more tightly clustered solution.

Metadata matrices were computed at each model order to quantify the impact of the BrainMap behavioral domains and paradigms for every network component. Hierarchical clustering was performed on these metadata matrices, and the cophenetic correlation coefficient was subsequently computed across the 19 dendrograms from *d* = 20–200. The observed CC*_c_* values are plotted in Figure 3, and ranged from a minimum of 0.438 to a maximum of 0.519, with a mean of 0.473 ± 0.020. ICA model orders corresponding to the two highest CC*_c_* values were identified at *d* = 20 (*CC_c_* = 0.5119) and *d* = 70 (*CC_c_* = 0.5198), while *d* = 50 and *d* = 190 networks were observed to correspond to the lowest CC*_c_* values. The dendrograms for these two highest (i.e., more clear distribution of functional properties for ICNs) and two lowest (i.e., less interpretable division of functional properties for ICNs) decompositions are shown in Figure 4. The pairwise relationships between any two variables (i.e., metadata classes) are illustrated, where each branch of the dendrogram represents an individual metadata class (i.e., behavioral domain or paradigm) in the BrainMap database. In general, the dissimilarity scale, or y-axis, of a dendrogram ranges from 0 to 1 where lower branching points indicate high similarities among clusters and are thus most desirable. Visual inspection of these graphs indicates that the two dendrograms with the most branching points low on the dissimilarity scale exemplify a good clustering solution that fit the metadata matrices well, while the two dendrograms with higher branching points do not, in agreement with the more quantitative differences in CC*_c_* values.

### Intrinsic connectivity network fractionation

**Figure 5:**
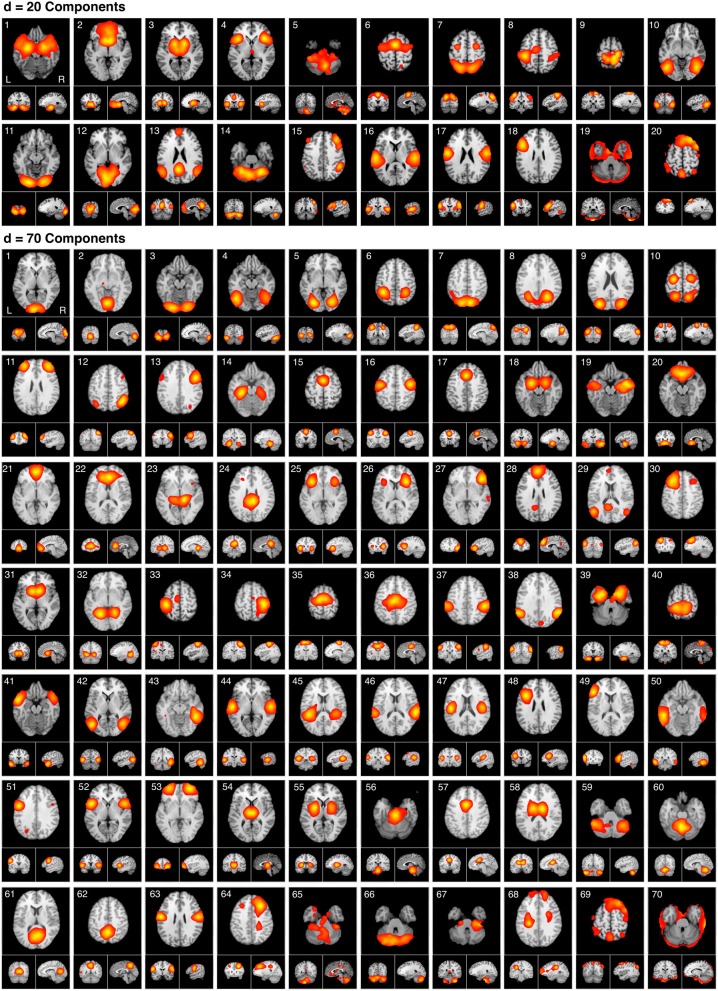
**The spatial topography of ICA model orders yielding high quality decompositions identified by producing the highest CC*_c_* values at *d* = 20 and *d* = 70**. The *d* = 20 components are presented in the same order as provided in Laird et al., 2011a. The *d* = 70 components mirror the hierarchical network organization of their respective model order, beginning with the most similar components followed by the least similar. ICA maps were converted to z statistic images via a normalized mixture model fit, thresholded at *z* > 4, and viewed in standard (Talairach) brain space. Orthogonal slices of the representative point in space are shown for each component.

Our results indicated that high quality decompositions were obtained at ICA model orders of *d* = 20 and *d* = 70, as these yielded CC*_c_* values that were two standard deviations higher than the mean. Thus, we sought to investigate general trends in the functional significance of how BrainMap-based ICNs fractionate from large-scale networks to smaller sub-networks specifically as a result of increasing model order from *d* = 20 to *d* = 70. The spatial topographies of these ICA components are shown in Figure 5. HCA was performed on the metadata matrices to determine similarity across behavioral domains and paradigms; in this stage, we transposed the matrices and repeated the analysis to quantify similarity across networks at each of the two best-fit model orders, similar to the procedure developed by Laird et al. (2011a).

**Figure 6:**
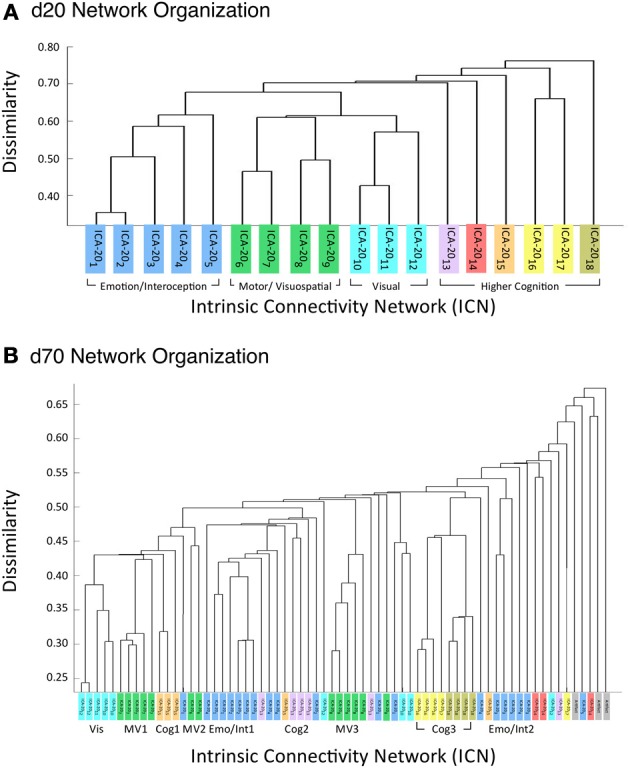
**HCA of BrainMap-based ICNs at *d* = 20 and *d* = 70 provide insight into how low-model order networks fractionate into high-model order sub-networks. (A)** Network clustering of the *d* = 20 decomposition revealed three clear groupings of highly similar networks: emotional and interoceptive networks (blue), motor and visuospatial networks (green), and visual networks (cyan). The remaining components were associated with higher cognitive processes (warm colors). The cognitive networks were behaviorally dissimilar across components and other network groups, as indicated by high branching points in its dendrogram. **(B)** Network clustering of the *d* = 70 decomposition exhibited generally similar network organizational properties with the *d* = 20 dendrogram, but included subtle fractionation properties indicative of non-homogenous behavioral functions. The left–to–right ordering of networks in the above dendrograms are the same as presented in Figure 5. Vis, visual; M/V, motor and visuospatial; Cog, cognitive; Emot/Int, emotional and interoceptive.

Figure 6A illustrates the behavioral network groupings at *d* = 20 where each dendrogram branch corresponds to an individual non-artifactual ICA network. Visual inspection of dendrogram indicated that the decomposition included three clear groupings of networks that were highly similar in terms of their behavioral metadata (not their spatial topography), and one set of dissimilar networks (seen on the far right). Previous HCA results revealed that these four *d* = 20 network groupings can be classified on the basis of their associated mental processes: (1) emotional and interoceptive processes that included networks for limbic and medial temporal areas, subgenual anterior cingulate cortex (ACC) and orbital frontal cortex (OFC), bilateral basal ganglia and thalamus, bilateral anterior insula and anterior cingulate cortex (blue networks; ICA-20_1_ − ICA-20_5_); (2) motor and visuospatial integration, coordination, and execution that included premotor and supplementary motor cortices, DLPFC and posterior parietal cortices, hand areas of the primary sensorimotor cortices, and superior parietal lobule (green networks; ICA-20_6_ − ICA-20_9_); (3) visual perception, including visual association cortices, as well as lateral and medial posterior occipital cortices (cyan networks; ICA-20_10_ − ICA-20_12_); and (4) higher cognitive processes that included the default mode network, cerebellar network, right-lateralized fronto-parietal cortices, auditory cortices, mouth areas of the primary sensorimotor cortices, and left-lateralized fronto-parietal cortices (warm-colored networks; ICA-20_13_ − ICA-20_18_). The last two ICA components (ICA-20_19_, ICA-20_20_) were deemed to be artifacts characterized by uniform metadata distributions (Laird et al., 2011a).

Figure 6A illustrates that the motor and visuospatial networks were found to be more similar to the visual perception networks than to the emotional and interoceptive networks, as indicated by their different branching heights. In addition, the divergent cognitive networks were observed to be strongly dissimilar across components, compared to each other as well as to other groupings of networks. Visual inspection of the dendrogram observed at *d* = 70 (Figure 6B) exhibited grossly similar clustering structure to that of *d* = 20, but included subtle organizational differences indicative of functional network fractionation properties. We employed the *fslcc* tool available in FSL (Jenkinson et al., 2012), as a means to track network fractionation from 20 components to 70 components. Cross-correlation values were computed between every network in the *d* = 20 decomposition with every network in the *d* = 70 decomposition. A high correlation value indicated a strong correspondence between spatial topographies across model order. Using these values, each high-model order network was grouped with its highest corresponding low-model order “parent” network, with exception to 3 artifactual networks characterized by implausible activation patterns. As seen by the color-coding scheme defined for the *d* = 20 networks, the majority of *d* = 70 networks in Figure 6B remained clustered with networks matching their original *d* = 20 group, but these groups were fractionated into multiple smaller sub-groups, suggesting fractionation as a result of non-homogenous behavioral functions. Figure 6B illustrates how the visuomotor networks (green) were split into three separate sub-groups of varying numbers of networks (MV1, MV2, MV3), while the emotional and interoceptive networks (blue) were divided into two sub-groups (Emo/Int1, Emo/Int2). The higher cognitive networks (warm colors) were fractionated into three sub-groups (Cog1, Cog2, Cog3), while the visual networks (cyan) remained intact and were not sub-divided (Vis).

### Visuomotor fractionation

**Figure 7:**
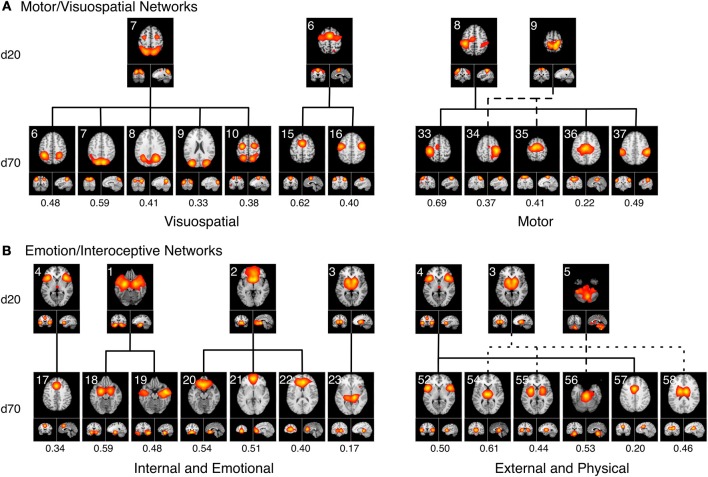
**Topological fractionation of select BrainMap-based ICNs are shown from low (*d* = 20) to high (*d* = 70) model orders**. The spatial cross correlation between the *d* = 20 and *d* = 70 networks are indicated below each respective *d* = 70 network. **(A)** The Motor/Visuospatial networks, which were grouped into one cluster at *d* = 20 (green ICNs in Figure 6), split into three separate clusters at *d* = 70. Metadata associated with these ICNs indicated that two clusters were more closely related to visuospatial tasks while the other cluster was highly linked motor tasks. **(B)** The Emotion/Interoception networks (blue ICNs in Figure 6) showed a more complex fractionation at *d* = 70 when splitting into two main clusters. Both of these clusters contained sub-networks from nearly all of their *d* = 20 ICNs; however, the metadata associated with these *d* = 70 ICNs showed a clear division in functional characterization into an initial cluster linked to internal and emotional processes and another cluster linked to external and physical processes.

Figure 7A delineates how the visuomotor networks at *d* = 20 (ICA-20_6_, ICA-20_7_, ICA-20_8_, ICA-20_9_; green) split into three distinct and non-overlapping sub-groups for separate motor and visuospatial processes at *d* = 70. According to this higher model order decomposition, the networks that included the DLPFC and posterior parietal cortices (Figure 6B, cluster MV1) and the premotor and supplementary motor cortices (Figure 6B, cluster MV2) were shown to exhibit strong visuospatial properties and thus found to be more similar to the visual perception networks. These visuospatial tasks included saccades/anti-saccades, mental rotation, visual pursuit and visual distraction. In contrast, the networks associated with motor processing were clustered into a different group (Figure 6B, cluster MV3). These included sub-networks of the hand areas of the primary sensorimotor cortices and superior parietal lobule, and were generally associated with finger tapping, flexion/extension, pointing, and grasping.

### Emotional and interoceptive fractionation

A strong dichotomy was present within the emotional and interoceptive sub-networks at *d* = 70 as shown in Figure 7B. Instead of observing a simple splitting in which individual *d* = 20 networks (ICA-20_1_, ICA-20_2_, ICA-20_3_, ICA-20_4_, ICA-20_5_; Figure 6 blue) were represented in only one high-model order cluster, we observed a more mixed and complex degree of fractionation. The *d* = 20 emotion/interoceptive grouping of networks split into two main clusters at d = 70 in which both clusters were comprised of sub-networks from nearly all five of the original *d* = 20 components. Specifically, the *d* = 20 subcortical network (ICA-20_3_) fractionated into four different *d* = 70 sub-networks (including separate components for putamen, thalamus, and basal ganglia), which thematically were associated with tactile paradigms and interoceptive processes. Overall, examination of the functional nature of this complex dichotomy of emotional and interoceptive networks revealed one cluster of sub-networks (Figure 6B, cluster Emo/Int1) was associated with internal, emotional, and introspective behaviors requiring little muscle activity, such as emotional picture discrimination, deception, resting state, anxiety, and thirst stimulation paradigms. In contrast, the other cluster of sub-networks (Figure 6B, cluster Emo/Int2) was related to more external stimuli or behaviors requiring muscle control over physical functions, such as micturition and bladder tasks, pain, thermal and tactile stimulation, TMS, and action preparation.

### Cognitive fractionation

With respect to the higher cognitive networks (warm colors), we observed *d* = 70 segregation of networks into three distinct clusters. One sub-network grouping (Figure 6B, cluster Cog1) was associated with decomposition of the right-lateralized fronto-parietal network (ICA-20_15_) into three sub-networks, which relate to the behavioral functions of reasoning, attention, inhibition, and working memory. A second cognitive subgroup (Figure 6B, cluster Cog2) included one right-lateralized fronto-parietal sub-network and three sub-networks representing fractionated components of the default mode network (ICA-20_13_), associated with theory of mind and social cognition tasks. Lastly, the *d* = 20 networks for auditory, speech, and language processes (ICA-20_16_, ICA-20_17_, ICA-20_18_, respectively) segregated into the same cluster at *d* = 70 (Figure 6B, cluster Cog3), preserving functional groupings seen at the lower model order.

### Visual fractionation

In contrast to the extensive splitting of the visuospatial, emotional/interoceptive, and cognitive network groupings, the visual networks (ICA-20_10_, ICA-20_11_, ICA-20_12_; cyan) remained highly similar and relatively intact across model orders (Figure 6B, clust Vis), with some minor splitting of single components at other locations in the dendrograms that were related to correspondences between the visual association areas with higher cognitive functions.

## Discussion

Previously, we demonstrated that the combined application of ICA and hierarchical clustering analysis (HCA) to task activation patterns archived in the BrainMap database identify and guide functional interpretation of intrinsic connectivity networks (Laird et al., 2011a). This has been shown for a standard low model decomposition (i.e., *d* = 20). Here, we applied the same methodology at a range of model orders from *d* = 20–200, in intervals of 10, to assess the effects of ICA dimensionality. We used the cophenetic correlation coefficient as a general statistic to characterize how distributions of functional properties fit brain networks across a range of decompositions. In doing so, we identified two model orders that provide the most interpretable segregation of BrainMap behavioral domain and paradigm labels, thereby maximizing the ability of behavioral meta-data to inform co-activation networks in the BrainMap context. Moreover, we found that this network-based clustering method provided insight into how large-scale networks fractionate into finer sub-networks when transitioning from low order decompositions (e.g., *d* = 20) to higher order decompositions (e.g., *d* = 70).

### Model order selection and agreement with resting state data

The cophenetic correlation coefficient was found to vary as model order was increased or decreased. Each model order results in different patterns of network spatial topographies, which influences the weightings that correspond to how strongly each behavioral domain or paradigm is associated with the ICA components. The resultant metadata matrices subsequently vary across model order, yielding dendrograms than can be differentiated in terms of their goodness of fit to the clustered data matrices. Our findings suggest that the 20- and 70-component ICA decompositions of BrainMap co-activation networks, when complemented with their metadata for functional interpretation, yielded more informative networks than other model orders in the tested range.

We do not assert that we have unequivocally proven that, for example, a model order of 50 is “superior” compared to a model order of 20. On the contrary, examination of the spatial topographies and associated metadata for networks across all model orders tested were found to be interpretable. Furthermore, we believe that rather than focus on a single decomposition, multiple iterations of ICA at different model orders are necessary to truly understand the complexity of intrinsic connectivity networks in both task-based and resting state neuroimaging data. That is, we endorse the practice of performing analyses that include decompositions across multiple model orders in order to draw more meaningful network inferences. However, the full and complete assessment of network fractionation across 19 decompositions (2090 total ICA components) is simply impractical. At this time, we lack the summary statistics and visualization capabilities for disseminating that level of data complexity. Thus, our goal in the present study was to provide an approach to identify a subset of model orders to facilitate cross-decomposition comparisons via a quantitative clustering-based metric. We intend to utilize these results to guide the next stage of our investigation of fractionation of BrainMap co-activation networks, which will focus on a more highly detailed explication of sub-network functional organization at *d* = 70. Given the results of the present study, we are less likely to pursue further analyses at *d* > 120; although, there is some interest in assessing if model fit continues to decline past *d* = 200.

Notably, previous studies investigating ICA model order of resting state fMRI data indicated similar results recommending 20 components for a low model order decomposition and around 70 components for a high model order decomposition (Abou Elseoud et al., 2010, 2011). Abou Elseoud et al. (2010) reached this conclusion by evaluating various topological properties and consistency measures (e.g., volume, mean *z*-score, stability, repeatability) of ICA decompositions across a wide range of model orders. We did not initially anticipate that our metric for more interpretable model orders of task-based co-activation networks would yield identical results to that for resting state networks. However, in light of the strong correspondence between spatial topographies across these data sets, it is reasonable to observe similar correspondence in model order considering that it has been posited that resting state networks do reflect functional, task-based networks (Fox and Raichle, 2007; Smith et al., 2009).

One notable difference was observed between the present study and the related work by Abou Elseoud et al. (2010). In their results, they observed significant increases in *z*-score only up to model order 80, while our results revealed a linear increase in *z*-score values up to the *d* = 200 decomposition. This may reflect an aspect of data features wherein BrainMap task activation patterns no longer behave similar to resting state FMRI data. Our approach of blurring thousands of reported stereotactic coordinates of activation locations to create pseudo-activation images results in large, sparse data sets, which may be more favorable than BOLD FMRI for high model order ICA decompositions. At high dimensionalities, ICA tends to over-fit BOLD FMRI time-series data, in which the noise inherently associated with FMRI signal overpowers the true physiological neuronal co-oscillations (Li et al., 2007; Abou Elseoud et al., 2010). Thus, in addition to the number of studies employed in our analyses, task-activation-derived ICNs may be intrinsically more powerful and capable of finer division (i.e., higher model orders) than resting state derived ICNs.

### Consistency of brainmap-based ICNs

A number of factors might have influence on the spatial topography of resultant ICA decompositions, which would affect further clustering and functional interpretation conclusions.

Thus, we performed a number of additional analyses to examine the consistency of our results. First, we performed ICA (*d* = 20) on the same 8,637 experiments in a randomized order. Using a spatial cross correlation analysis, the original ICNs and the randomized ICNs were nearly identical, indicating that the spatial maps are not dependent upon the order or experiment input. Furthermore, varying the FWHM of the Gaussian parameter by applying the Eickhoff et al. (2009) smoothing algorithm when creating the 8,637 activation images resulted in ICNs with similar topography. In terms of clustering approaches, we investigated the use of other linkage methods (e.g., *average* linkage) and observed consistent similar groupings of networks and overall higher CC*_c_* values with similar CC*_c_* peaks. However, the results of using this algorithm showed very little variance in CC*_c_* values (mean = 0.68 ± 0.009) across model orders, indicating that it is less sensitive to model order.

**Figure 8:**
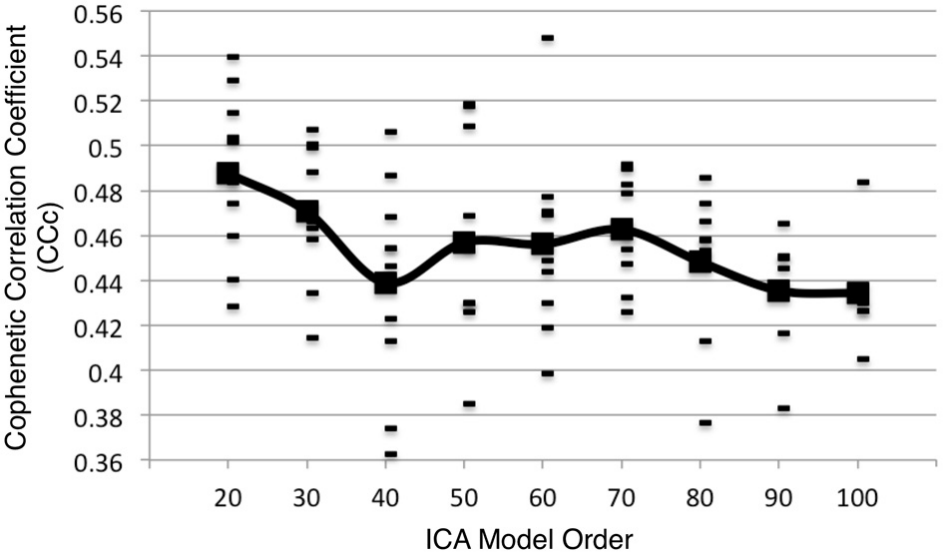
**Additional analyses were performed on 10 random subsets of 90% of the experiments in the BrainMap database at the time of the initial analysis following the same procedure as outlined in Figure 1 (up to *d* = 100)**. The CCc values resulting from the 90% subsets follow a similar trend as shown in Figure 3 when averaging across all subsets.

Additional analyses were performed on 10 random subsets of 90% of the experiments in the BrainMap database at the time of the initial analysis. Following the same procedure as outlined in Figure 1 up to *d* = 100, the peaks in the CC*_c_* values initially observed at *d* = 20 and *d* = 70 components continued to be observed in 10 random subsets of 90% when averaging across all samples, although the observed peak at *d* = 70 was not as robust as the peak at *d* = 20, as the results were somewhat plateaued from *d* = 50 to 70 (Figure 8). Overall, results from these additional analyses followed a similar trend as shown in Figure 3, and suggest that *d* = 20 and *d* = 70 exemplify appropriate model orders for further functional interpretation in BrainMap-based ICN analyses.

Lastly, we acknowledge the possibility that substantial changes in the selection of published studies archived in BrainMap would result in relatively different decompositions of ICNs depending on the cognitive aspects of experiments added to the sample (e.g., a large increase in interoceptive experiments would produce an ICA decomposition more weighted toward ICNs associated with interoceptive processes).

### Intrinsic connectivity network fractionation

Comparing dendrograms for low- and high-model order decompositions (Figure 6) allowed visual conceptualization of the differences between networks associated with homogenous and heterogeneous functions. Generally, low-model order networks associated with low-level perceptual function showed little breakdown into sub-networks at a higher model order, such as for visual and auditory perception. However, multimodal networks associated with higher-level processes tended to display a high amount of fractionation into multiple sub-networks according to breakdown of their constituent functions. Similar results have previously been found in high-model order ICA decompositions of resting state FMRI, in agreement with this result (Kiviniemi et al., 2009; Smith et al., 2009; Abou Elseoud et al., 2010, 2011).

Overall, we observed that the 3 *d* = 20 visual networks were subdivided into 9 sub-networks at *d* = 70, 4 visuomotor networks were fractionated into 13 sub-networks, 5 emotional and interceptive networks into 20 and 6 cognitive networks into 23 sub-networks. This translates to an average of 3 *d* = 70 sub-networks for every visual network, 3.25 for visuomotor, 4 for emotional/interoceptive, and 3.83 for cognitive, which suggests that for these exemplar model orders, the emotional and interoceptive networks were found to fractionate at the highest rate. When comparing fractionation properties from *d* = 20 to *d* = 70 networks across groupings, we found the visuomotor network split into non-overlapping visual and motor sub-networks to be clear and reasonable. However, the emotional and interoceptive splitting into 2 clusters that included contributions from nearly all original *d* = 20 networks will require more examination to fully understand. For this fractionation, the separation between sub-groups was comparatively increased in terms of distance along the dendrograms x-axis, which likely reflects a stronger dissimilarity and more fundamental dichotomy between emotional and interoceptive processes.

Lastly, the cognitive networks differed from both the visuomotor and the emotional/interoceptive fractionation schemes in that complex sub-groups were observed, but demonstrated fewer overlapping contributions than the emotional/interoceptive sub-networks. In comparison to the *d* = 20 organization, we found that many cognitive *d* = 70 sub-networks remained highly dissimilar, with the exception of the relatively homogenous and intact grouping for speech, audition, and language (Figure 6B, cluster Cog3). The right-lateralized fronto-parietal sub-networks (Figure 6B, cluster Cog1) were observed to split from the cognitive networks which were grouped with visual and visuospatial sub-networks, thus indicating greater homogeneity in functions associated with visual attention and reasoning. Similarly, the default mode sub-networks were segregated from their *d* = 20 cognitive neighbors and more strongly associated with emotional and introspective behaviors at *d* = 70 (Figure 6B, cluster Cog2). In turn, there was a group of strongly interoceptive sub-networks related to bladder control and external stimulation that were fractionated from their original emotional grouping at *d* = 20 to become more highly dissimilar to other sub-groups (Figure 6B, cluster Emo/Int2), thus positioning them near the remaining dissimilar cognitive sub-networks. These dendrogram shifts between *d* = 20 and *d* = 70 illustrate the functional impact of model order selection in ICA-based investigations of task co-activation networks.

### Use recommendations and future directions

The network images and associated BrainMap metadata generated in this study have been made available for download (brainmap.org/icns) to serve as a shared resource for the neuroscience community. Currently BrainMap ICA components are provided at dimensionalities of 20 and 70, which may useful in the interpretation of the functional significance of future resting state results. For resting state networks that are not a close match with the 20- or 70-dimension BrainMap networks, the Mango/BrainMap behavioral analysis tool can be used to identify significant behaviors within a ROI or network mask (Lancaster et al., 2012). Additionally, BrainMap ICNs could serve as seed regions or masks for future resting state analyses in efforts to avoid the inherent problems associated with double dipping (Kriegeskorte et al., 2009). Future work in this area will involve a thorough analysis of BrainMap-based ICNs at additional model orders to provide better insight to the hierarchical organization of these functional networks.

## Limitations

The results observed in the present study are based on the premise that the cophenetic correlation coefficient of metadata matrices provides a meaningful metric to discriminate between ICA model orders. We based this assertion on the fact that the CC*_c_* measures fit between matrix data and corresponding clustering solution. However, this procedure is highly dependent on BrainMap's taxonomy, including the design and implementation for how BrainMap personnel manually code studies from the literature. This is most critical for the metadata fields of behavioral domain and paradigm, since our study is based on these taxonomic classifiers.

These results are additionally dependent on the heterogeneity of experiments in the BrainMap database. There is an uneven distribution of experiments archived in BrainMap in which 74% of experiments elicit cognitive processes, 26% are emotion related processes, 23% of experiments are perception related, 21% are associated with action paradigms, and 3% with interoceptive fields. It is not uncommon for these experiments to be coded with more than one behavioral domain, thus explaining for the proportions of behaviors summing greater than 1.

Since the present results are thus limited to the current instantiation of the BrainMap database, future work may involve replication of the results following extension and/or refinement of the BrainMap metadata taxonomy. The importance of functional neuroimaging ontologies cannot be underestimated, as evidenced by this and other studies. Currently, statistical methodologies for large-scale meta-analysis and data mining investigations are being developed for the BrainMap Project. But even with excellent analysis strategies, meta-analytic results are critically affected by standards and conventions for neuroinformatics and psychoinformatics. Efforts to improve the BrainMap ontology are currently underway and likely will have an impact on the functional interpretation of not only task co-activation networks, but also functional brain networks derived from meta-analytic connectivity modeling (Laird et al., 2009; Eickhoff et al., 2010; Robinson et al., 2010; Zald et al., 2012) and connectivity-based parcellation (Eickhoff et al., 2011; Bzdok et al., 2013; Cieslik et al., 2013). Alternatively, a similar analysis could be carried out in a different task-based meta-analysis database of neuroimaging results, such as NeuroSynth (http://neurosynth.org; Yarkoni et al., 2011). Although recent text-based topic modeling of NeuroSynth data has yielded similar intrinsic connectivity networks to those seen here (Poldrack et al., 2012), to our knowledge the issue of model order has not yet been addressed in this context.

## Conclusion

We investigated the effects of BrainMap metadata distribution of functional properties across intrinsic connectivity networks as a method for identifying model orders that provide the most interpretable segregation of BrainMap behavioral domain and paradigm labels, thereby maximizing the ability of behavioral meta-data to inform BrainMap based co-activation networks. Results of our analyses indicated that ICA performed at a model order of *d* = 20 and *d* = 70 provides network metadata matrices with increased CC*_c_* fit. At *d* = 70, we found that the emotional and interoceptive networks fractionated at the highest rate into the largest number of sub-networks. We observed complex fractionation properties for cognitive and emotional/interoceptive networks and relatively simpler fractionation for visuomotor processes, while the visual perception networks remained relatively intact. These results suggest that selecting ICNs from a single model order may provide limited information, while interpreting ICNs across multiple model orders yields more dynamic information about the functional organization and hierarchical modularity of the human brain.

### Conflict of interest statement

The authors declare that the research was conducted in the absence of any commercial or financial relationships that could be construed as a potential conflict of interest.
